# In Vitro Study of Cerebrospinal Fluid Dynamics in a Shaken Basal Cistern after Experimental Subarachnoid Hemorrhage

**DOI:** 10.1371/journal.pone.0041677

**Published:** 2012-08-01

**Authors:** Ulrich Kertzscher, Torsten Schneider, Leonid Goubergrits, Klaus Affeld, Daniel Hänggi, Andreas Spuler

**Affiliations:** 1 Biofluid Mechanics Laboratory, Charité – Universitätsmedizin Berlin, Berlin, Germany; 2 Department of Neurosurgery, Heinrich-Heine-University, Düsseldorf, Germany; 3 Department of Neurosurgery, Helios Klinikum Berlin-Buch, Berlin, Germany; St Michael’s Hospital, University of Toronto, Canada

## Abstract

**Background:**

Cerebral arterial vasospasm leads to delayed cerebral ischemia and constitutes the major delayed complication following aneurysmal subarachnoid hemorrhage. Cerebral vasospasm can be reduced by increased blood clearance from the subarachnoid space. Clinical pilot studies allow the hypothesis that the clearance of subarachnoid blood is facilitated by means of head shaking. A major obstacle for meaningful clinical studies is the lack of data on appropriate parameters of head shaking. Our *in vitro* study aims to provide these essential parameters.

**Methodology/Principal Findings:**

A model of the basal cerebral cistern was derived from human magnetic resonance imaging data. Subarachnoid hemorrhage was simulated by addition of dyed experimental blood to transparent experimental cerebrospinal fluid (CSF) filling the model of the basal cerebral cistern. Effects of various head positions and head motion settings (shaking angle amplitudes and shaking frequencies) on blood clearance were investigated using the quantitative dye washout method. Blood washout can be divided into two phases: Blood/CSF mixing and clearance. The major effect of shaking consists in better mixing of blood and CSF thereby increasing clearance rate. Without shaking, blood/CSF mixing and blood clearance in the basal cerebral cistern are hampered by differences in density and viscosity of blood and CSF. Blood clearance increases with decreased shaking frequency and with increased shaking angle amplitude. Head shaking facilitates clearance by varying the direction of gravitational force.

**Conclusions/Significance:**

From this *in vitro* study can be inferred that patient or head shaking with large shaking angles at low frequency is a promising therapeutic strategy to increase blood clearance from the subarachnoid space.

## Introduction

Despite the elaborate current treatment strategies, cerebral arterial vasospasm leading to delayed cerebral ischemia constitutes the major delayed complication following aneurysmal subarachnoid hemorrhage (SAH). Vasospasm is associated with significant morbidity and mortality rates [1]. Independent predictors of cerebral vasospasm are clot volume in the subarachnoid space [2], [3] and clot clearance rate [4]. Being a function of the amount of subarachnoid blood, the incidence of vasospasm can be reduced by increasing blood clearance from the basal cerebral cisterns. This can be achieved by means of increased perfusion of the subarachnoid space with CSF alone [5] or combined with intracisternal thrombolysis [6].

Clearance of cisternal blood may also be facilitated by repeated head motion. Two different head motion strategies, translational movement [7], [8] and bidirectional rotation [9], have been applied clinically. Since in these studies head motion was combined with an intensified lumboventricular lavage [9], the contribution of bidirectional rotation to the clearance of the subarachnoid space is difficult to dissect from the effect of increased CSF flow. Furthermore, none of the studies of head motion investigated variations of head shaking parameters.

The aim of our study was to define clinically applicable settings for effective head shaking. Using a quantified dye washout technique *in vitro*, we visualized and analyzed CSF flow and cisternal blood clearance in the central basal cistern. In order to understand the basic blood washing process in the basal cistern, we investigated the clearance of a blood model during head shaking with a steady net CSF flow.

## Materials and Methods

### Phantom Model of the Basal Cistern

**Figure 1:**
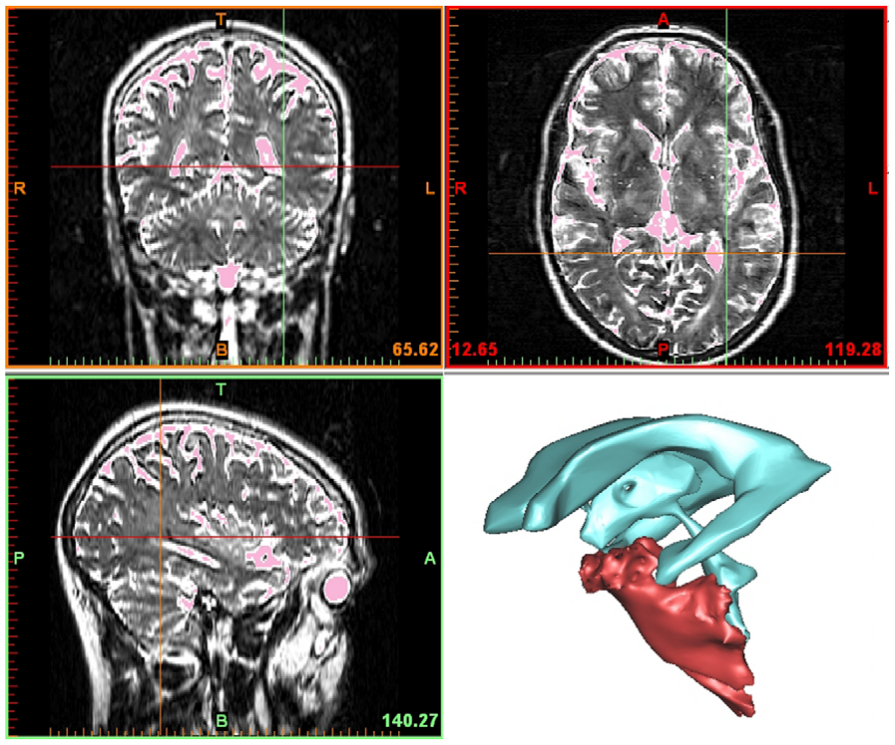
Anatomical data of the model cistern. Three orthogonal slices of the MRI data with cerebrospinal fluid in the cerebral sulci, ventricles, and basal cisterns indicated in pink. The three-dimensionally reconstructed basal cistern (red) and the ventricular system (blue) are shown for a better space orientation.

The study complies with the declaration of Helsinki. The study was done in a model constructed from human magnetic resonance imaging data of the brain. All data are from one single human who is co-author (DH) and gave written voluntary informed consent. Approval was given by the ethic committee of the Heinrich-Heine-University Düsseldorf, Germany. The model of the basal subarachnoid cistern consists of the prepontine cistern, the pontocerebellar cistern, and the pentagonal cistern. It was fabricated using a stereolithography (STL) geometry. Magnetic resonance imaging (MRI) data were acquired from the above mentioned healthy 35-year-old male volunteer using a 1.5 T scanner (Magnetom Avanto™, Siemens, Erlangen, Germany) by means of a spin echo (SE) sequence (sequence variant SK) with a slice thickness of 2.4 mm, a repetition time (TR) of 4,000.0 ms, and an echoing time (TE) of 250.0 ms. Data acquisition resulted in a stack of 180 slices with a voxel resolution of 0.82 mm×0.82 mm×1.2 mm. Three-dimensional segmentation of the subarachnoid cistern was done using the software Mimics™ (Materialise NV, Leuven, Belgium) (Figure 1).

**Figure 2:**
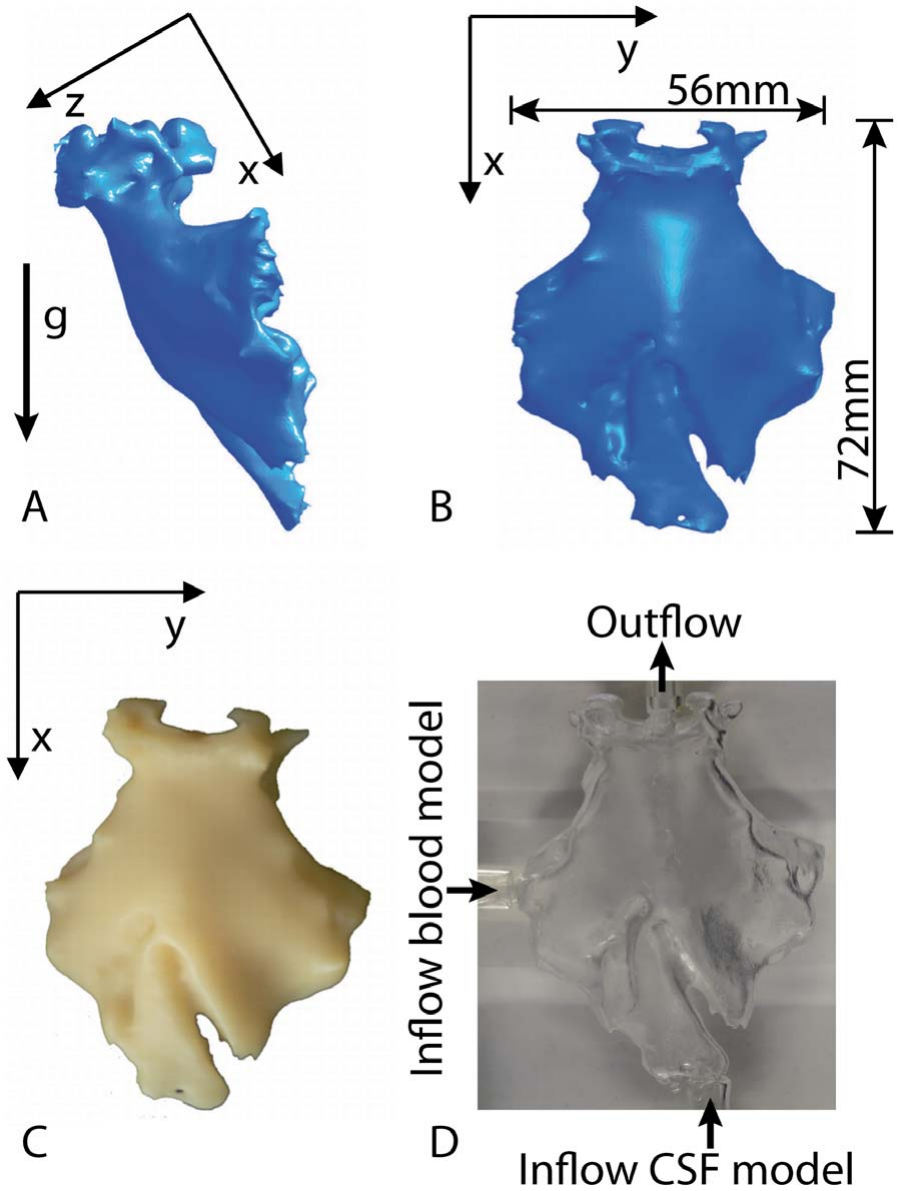
Model of the basal cistern. Surface of the reconstructed basal cistern in a lateral (A) and frontal (B) view. The g-arrow indicates the longitudinal axis of the human body which is aligned with the gravitational force for a sitting patient. Photo (C) shows the corresponding wax model and photo (D) the fabricated silicone model. Arrows indicate the sites of the inflow of the blood model as well as the inflow and outflow of the CSF model.

Segmentation was performed manually based on boundaries defined primarily by automatic segmentation using intensity signal thresholding. Finally, the reconstructed geometry was converted into a STL file format. This geometry served as physical model in our experimental study (Figure 2A and B). The volume of the experimental basal cistern was 20 ml. Sizes of the cistern (length and width) are indicated in Figure 2B. Mean depth of the cistern was approximately 10 mm.

From the reconstructed surface geometry (STL file) a 1∶1 wax cast was fabricated by means of a rapid prototyping printer thermojet (PORTEC, Zella-Mehlis, Germany) with a spatial resolution of 0.1 mm (Figure 2C). Thereafter, a transparent silicone phantom model of the basal cistern was generated as block cast. For this, the wax model with ducts representing the CSF inflow and outflow tracts was embedded in a mixture of Elastosil™ RT 601 A and B (Wacker Chemie, Munich, Germany). Inflow and outflow tracts were mounted along the longitudinal axis of the cistern model at the narrowest regions of the boundaries of the reconstructed basal cistern geometry. Inside diameters of inflow and outflow tracts were 4 mm each. The diameters were selected according to the cross-sectional areas at the respective regions (basal cistern inflow/outflow) identified on the MRI data. The wax was melted out in a furnace at 150°C resulting in the transparent silicone model of the basal cistern (Figure 2D).

### Experimental Setup

**Figure 3:**
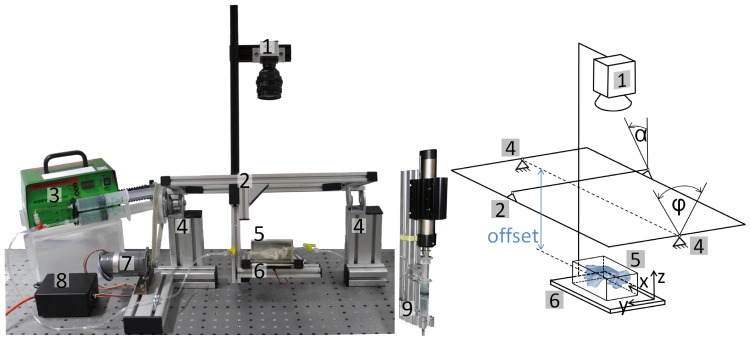
Experimental setup. Left: Photo of the experimental setup mounted on the optical table: (1) CCD camera; (2) rigid frame mounting the basal cistern model (5), light source (6) and camera (1) on two pylons (4) allowing x-axis rotation; (3) syringe pump; (7) and (8) step motor with programmable controller; (9) pulse generator. Right: Schematic drawing of the experimental setup.

The study was performed in a custom-built experimental setup (Figure 3). The setup was designed to allow flow visualization simultaneously with simulated head shaking: light source, phantom model and camera were combined in a rigid rotatable construction. The axis of rotation (x-axis) parallel to the horizon and the (longitudinal) center of the cistern model were mounted with an offset of 0.15 m to the longitudinal axis of the basal cistern (head) model (Figure 3). The setup permitted an engine driven shaking with different speeds and different shaking angle amplitudes φ_max_. The assembly transmitting the drive mechanics into the shaking motion emulated sinusoidal deflection curves. Rotation of the cistern model around the y-axis (Figure 3, right) simulated three head (patient) positions: α = 0° – supine patient with slightly reclined neck and x-axis of the basal cistern parallel to the axis of rotation, α = 30° – supine patient with normal head position and x-axis of the basal cistern deviating 30° from the axis of rotation, and α = 90° – semi-sitting patient and x-axis of the basal cistern deviating 90° from the axis of rotation.

Experimental CSF was a mixture of distilled water with glycerin in a concentration of 43.7% (by weight) glycerin, whereas experimental blood was a mixture in a concentration of 70.0% (by weight) glycerin.

This specific composition of the mixtures offers three major advantages: First, experimental CSF and blood have the same density ratio (ρ_CSF_/ρ_blood_) of 0.95 as known for physiological CSF and blood [10]–[12] at a temperature of 37°C (ρ_CSF_ = 1.000 kg/l and ρ_blood_ = 1.048 kg/l). Second, the refractive indices of experimental CSF (n = 1.3891) and experimental blood (n = 1.4274) measured with the refractometer DR201-95™ (A. Krüss Optronic, Hamburg, Germany) sufficiently approximate the refractive index of silicone (n = 1.409, manufacturer information). Third, one hour of *in vitro* measurement corresponds to four hours *in vivo* taking into account the laws of dimensionless flow similarity. This accelerates the time-consuming tests. The same scaling is valid for the shaking frequency: a frequency of 10 rpm *in vitro* is equivalent to 2.5 rpm *in vivo*. *In vitro* time scaling was achieved by increasing viscosities and flow rates according to the dimensionless Reynolds (Re  =  U**.** L/ν) and Strouhal (St  =  L/U**.** T) numbers with U as characteristic (mean) velocity (m/s), L as characteristic length of the flow model (m), ν as kinematic viscosity of the test fluid (m^2^/s), and T as characteristic flow time (s). The resulting kinematic viscosities of experimental CSF and blood were 3.40·10^−6^ m^2^/s and 15.80·10^−6^ m^2^/s respectively at 24°C, the temperature of the fluids during experiments. Kinematic viscosities of the test fluids were measured by means of a SCHOTT Ubbelohde Viscometer™ 50101/0a and Ia (SCHOTT Instruments, Mainz, Germany) with a device accuracy of 1%. *In vivo* kinematic viscosities depend on various parameters (e.g. hematocrit, protein content and temperature) and were assumed to approximate 0.75·10^−6^ m^2^/s for CSF [13] and 3.50·10^−6^ m^2^/s for blood [14]. Thus, the ratio of CSF and blood viscosity is *in vitro* the same as *in vivo* (0.75∶3.50 and 3.40∶15.80).

The experimental setup allows also experiments simulating the pulsatile nature of CSF flow [15]. For this purpose a sinusoidal CSF flow was superimposed to the net flow at the inflow duct. The sinusoidal flow pulse was generated by means of a linear motor PS01-48×240F-C™ (LinMot™, NTI, Switzerland) attached to a 20 ml Original-Perfusor™ syringe (B. Braun, Melsungen, Germany) filled with experimental CSF (Figure 3). A sinusoidal pulse with an *in vitro* frequency of 4 Hz (corresponding 1 Hz *in vivo*) and an *in vitro* amplitude of CSF flow rate of 4 ml/s (1 ml/s *in vivo*) was used according to CSF flow curves observed in phase-contrast MRI measurements [16] and used in a numerical study of CSF flow [17].

### Dye Washout and Data Analysis

First, two reference experiments “CSF-washout” and “Blood-CSF-diffusion” were performed. The first experiment “CSF-washout” serves as the reference allowing a comparison of blood clearance with the dynamics of the CSF replacement without additional blood. The second reference experiment evaluates the impact of the diffusion between two fluid models (CSF and blood) affecting the kinematics of the CSF/blood mixing process. Second, experiments with eight different settings simulating blood washout were done (Table 1).

**Table 1:** Experimental parameters.

Exp.	1	2	3	4	5	6	7	8	9	10	11
**α (1**°**)**	0	0	0	0	0	0	30	90	0	0	0
**φ_max_ (1**°**)**	70	45	45	20	20	0	0	0	0	0	0
***f*** ** (rpm)** b	1	10	5	10	5	0	0	0	0	0	0
***f*** ** (rpm)** c	0.25	2.5	1.25	2.5	1.25	0	0	0	0	0	0

a CSF cerebrospinal fluid; Exp. experiment; α angle of the cistern model simulating head position; φ_max_ shaking angle amplitude;

*f* shaking frequency; rpm revolutions per minute;

b *in vitro* time scale;

c *in vivo* time scale.

Eight experimental settings with a steady physiological net CSF flow rate, settings of both reference experiments (Exp. 9 and 10), and experimental setting for pulsatile CSF flow (Exp. 11). Note that during Exp. 9 (“CSF-washout”) no blood was injected and during Exp. 10 (“Blood-CSF-diffusion”) zero CSF flow was simulated.^a^.

The CSF-washout reference experiment with experimental CSF alone i.e. without injection of experimental blood was performed as follows: The cistern model was filled with dyed experimental CSF. Dye in a concentration of 0.5 g/kg (100% of dye concentration) of the food coloring dye Patent Blue V™ (Schumann & Sohn, Karlsruhe, Germany) was used. A syringe pump (Perfusor Secura™, B. Braun, Melsungen, Germany) generated flow of non-dyed experimental CSF with a rate of 80 ml/h. The flow rate was calculated taking into account the physiological CSF net flow rate of approximately 20 ml/h [18] and Reynolds similarity.

The model was illuminated from the backside by diffuse light emitted from a custom-made panel of “cold white” light-emitting diodes (ASMT-QWBB™, Avago Technologies, San José, CA USA). Light intensity was adjusted to avoid overexposure and to utilize the full range of illumination dynamics (gray values between 0 and 255). Washout was recorded by a charge-coupled device (CCD) color video camera (UI 6250SE-C™, IDS Imaging Development Systems, Obersulm, Germany) with a resolution of 1600×1200 pixels, a manually adjusted constant white balance and an exposure time of 21 ms.

In experiments with SAH modeling, the basal cistern model was filled with non-dyed experimental CSF first. Thereafter, 10 ml of dyed experimental blood (0.5 g/kg dye concentration) was injected with a flow rate of 100 ml/h into the cistern model (Movie S1). This injection was generated by means of a syringe pump (R100EC™, Razel Scientific, St. Albans, VT USA) in order to minimize the variability of initial experimental conditions. Shaking and washout with non-dyed experimental CSF (flow rate of 80 ml/h) were started and recorded (Movie S2) simultaneously. Experimental setup and settings of the camera and light source were the same as in the reference experiments. *In vitro* data acquisition time was one hour (corresponding to four hours *in vivo*).

Image analysis by means of MATLAB™ (The MathWork, Natick, MA USA) consisted in converting color images into gray scale images (gray values ranging from 0 to 255), setting the region of interest (ROI), averaging grayscale values in the ROI for each image, normalizing (0–1) the gray values according to images with 100% and 0% dye concentration, and smoothing the results by averaging periods of 1 min recording. A normalized gray value of 1 represents light absorption of the model completely filled with undiluted dyed experimental blood (100% dye concentration). A normalized gray value of 0 was assigned to the absorption of the model filled with non-dyed experimental CSF. Normalized gray values of 50%, 25% and 12.5% experimental blood concentration were determined in additional experiments because of the non-linear relationship between normalized gray value and dye concentration.

The resulting curve of the “CSF-washout” reference experiment (Exp. 9) describes washout (replacement) of CSF. In the other experiments, the curves describe mixing of CSF and blood combined with blood washout. The dye washout process of CSF was characterized by two periods: half dye time, which is the period to achieve a gray value corresponding to a dye concentration of 50% in the calibration (normalized gray value of 0.90), and quarter dye time, which is the period between half dye time and time to achieve a gray value corresponding to a 25% of the dye concentration (normalized gray value of 0.81).

There is an important difference between the reference experiment and blood washout experiments: The reference experiment starts with a normalized gray value of 1, i.e. 100% of the dye concentration in 20 ml of CSF. The injection of 10 ml experimental blood with a dye concentration of 100% simulated SAH. In the case of full mixing between experimental dyed blood and non-dyed CSF, the resulting normalized gray value would correspond to 50% dye concentration or a normalized gray value of 0.90. Blood injection, however, does not result in full homogeneous mixing with CSF. In the worst case (no mixing), half of the model’s projected area (i.e. the ROI) represents dyed experimental blood (normalized gray value of 1) and the other half represents a non-dyed CSF model (normalized gray value of 0). In this case, the resulting mean normalized gray value (averaged over ROI) is equal 0.5.

After the blood washout was started, the mixing process first results in an increase of the normalized gray value meaning a homogenization of the blood/CSF mixture. Therefore, the curves for blood clearance are characterized by the mixing time T_M_ – the time during which the normalized gray value is increasing or constant, and by the clearance time T_C_ – the time between the end of mixing and the time to achieve a normalized gray value of 0.81 corresponding to a 50% reduction of the amount of blood (25% of dye concentration). In some cases this value was not reached during the experiment lasting one hour because the washout was so slow.

Additionally, we performed an experiment under pulsatile CSF flow conditions with settings corresponding to supine patient with slightly reclined neck (α = 0°) not treated with head shaking (Exp. 11).

### Dye Washout Experiments Repeatability

**Figure 4:**
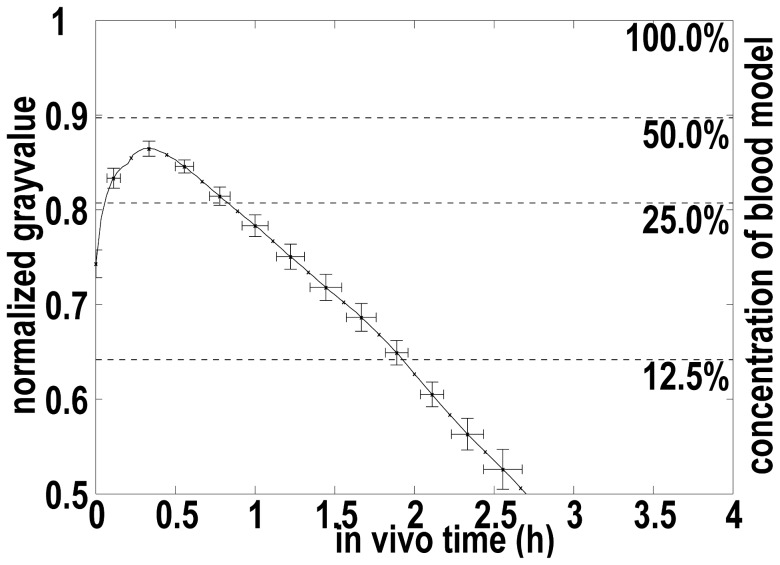
Repeatability of experiments. Standard deviation (n = 6) for normalized gray value and time to achieve a normalized gray value of Exp. 1 (see Table 1). The horizontal lines labeled with 100%, 50%, 25% and 12.5% represent the normalized gray values from a calibration experiment with according blood model concentrations.

In order to prove repeatability of the results in our experimental setup, we performed the dye washout experiment with one setting (Exp. 1, Table 1) six times. The high repeatability of our experiments illustrated in Figure 4 is characterized by the standard deviation of the gray values of less than 2%. This low variability of gray values results in slightly higher standard deviation (less than 9%) for the characteristic times defining the washout process. The high repeatability of the experimental setup was also proved in experiments with further three settings (Exp. 2, 4 and 6). These experiments were repeated 2 times each. The results (data not shown) confirmed the high repeatability observed in experiment with settings Exp.1.

## Results

**Figure 5:**
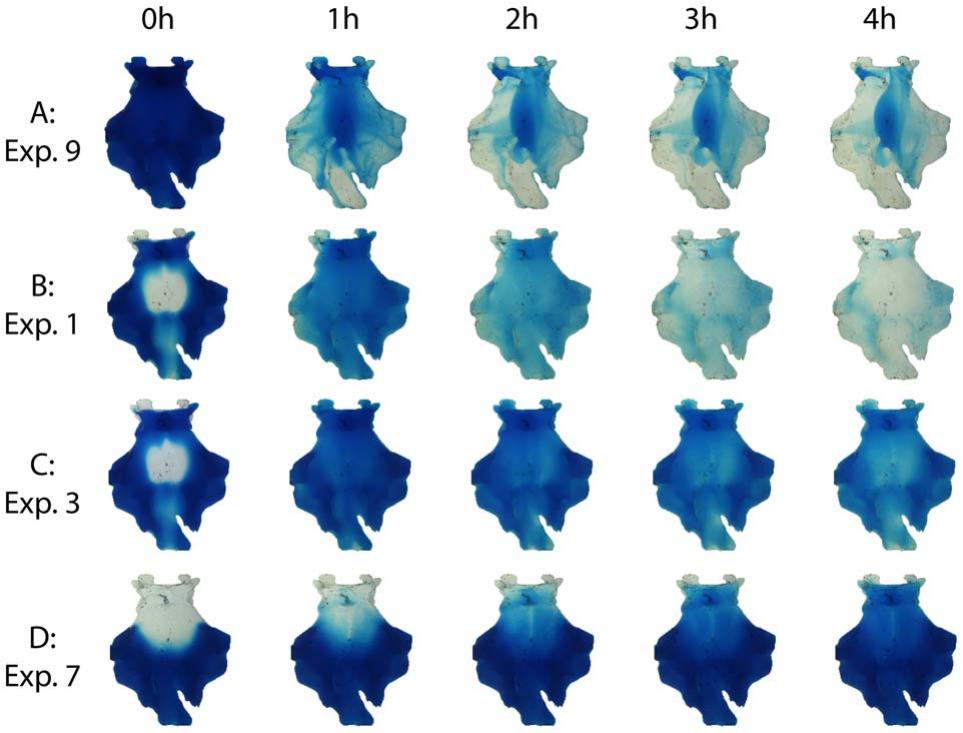
Examples of washout images at different time steps. Five *in vivo* time step images of four dye washout experiments. Settings of experiments are shown in Table 1.

**Figure 6:**
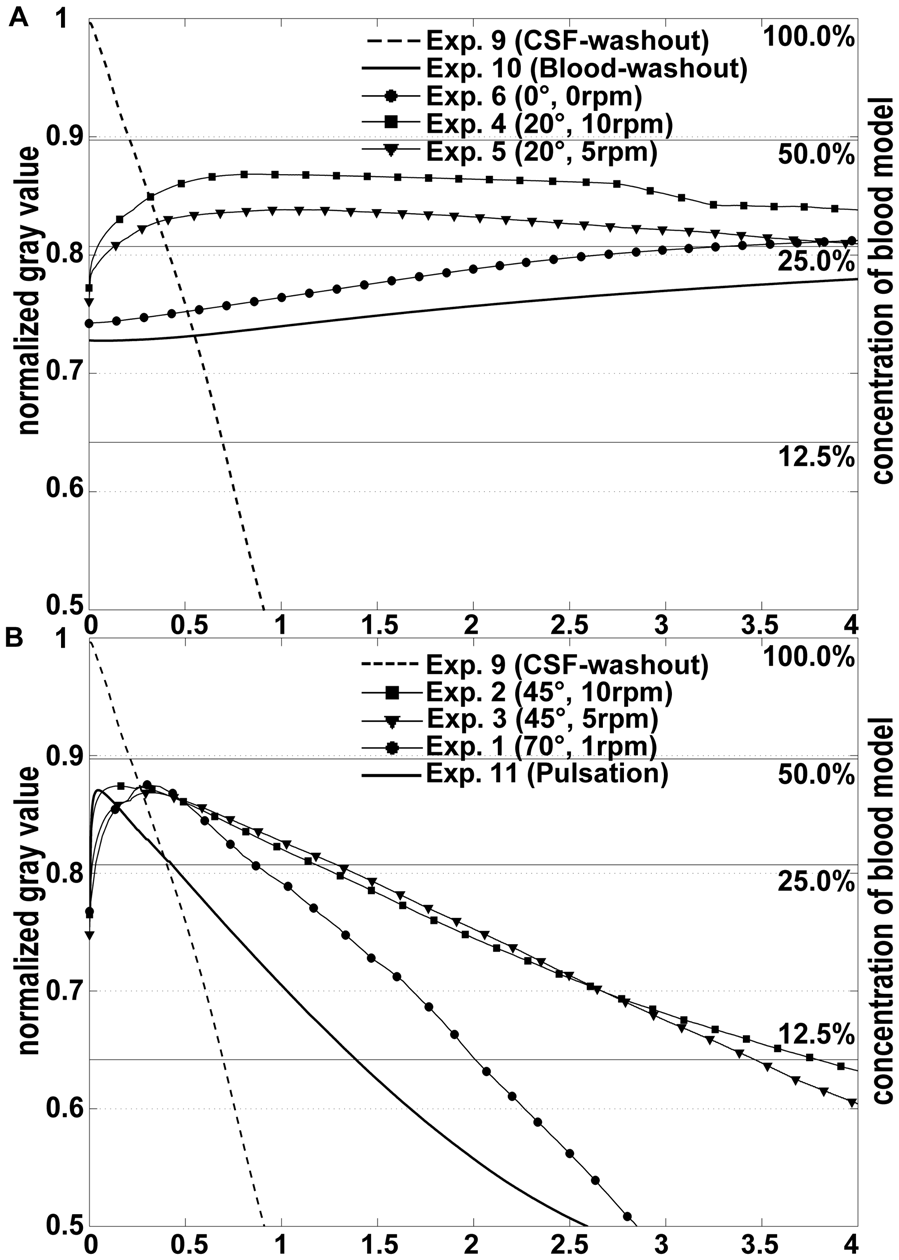
Time course of washout. Normalized gray value curves quantifying the washout process of six of the eight washout experiments, of both reference experiments and of the experiment with pulsatile conditions. For a better clearness graphs were displayed in two groups (A and B) with identical scales. The horizontal lines labeled with 100%, 50%, 25% and 12.5% represent the normalized gray values from a calibration experiment with according blood model concentrations.

Figure 5A shows the images of experimental CSF washout (Exp. 9) without blood for five time steps. This washout process is also described by the CSF washout graph in Figure 6. The *in vivo* half dye time in this experiment was 12.7 min, whereas the quarter dye period was 11.5 min (period between half dye time and time to achieve a gray value corresponding to the 25% of the dye concentration).

Three experiments (Exp. 6–8, Table 1) simulated blood washout without shaking (static washout) for three different head positions (cistern orientation α = 0°, 30° and 90°). Figure 5D shows an example of static blood washout for a cistern position of α = 30° (Exp. 7). In experiments 6 and 7, only mixing (resulting in increasing normalized gray values, see Figure 6A, curve of experiment 6) and no washout were observed in the experimental 60 min corresponding to 240 min *in vivo*. Only for a semi-sitting patient (cistern orientation α = 90°, Exp. 8), a very slow washout (clearance time T_C_ was not achieved) after a long period of mixing (*in vivo* T_M_ = 208 min) was observed.

Of all five experiments (Exp. 1–5, Table 1) simulating blood washout with shaking, washout was seen in the experiments with higher shaking amplitudes (see Figure 6B, Exp. 1–3) after a faster mixing process with a mean mixing time of T_M_ = 50±46.4 min. Table 2 summarizes mixing and clearance times for all five blood washout experiments simulating shaking.

**Table 2:** Mixing and clearance times.

Exp.	1	2	3	4	5	11
**T_M_, min**	19	13	19	83	51	3
**T_C_, min**	30	56	58	–	–	23

a Exp. experiment;

T_M_ mixing time;

T_C_ clearing time; min minutes.

*In vivo* times of mixing and clearance in all five experiments simulating blood washout with head shaking (Exp. 1–5) and experiment with pulsatile flow without head shaking (Exp. 11)^a^.

In the two experiments (settings Exp. 4 and 5) with a small shaking angle (φ_max_ = 20°) only a minor washout of experimental blood (clearance time was not achieved) was observed during an *in vivo* period of four hours. The mixing times of these two experiments (83 min and 51 min) were substantially higher than in the three experiments with higher amplitudes (mean T_M_ = 17±3.5 min).

Figure 5 (B and C) shows serial images of two washout experiments with higher amplitude shaking. In experiment 1 (Figure 5B and Movie S2), the shaking frequency was 1 rpm *in vitro* (corresponding to 0.25 rpm *in vivo*) and the shaking amplitude was φ_max_ = 70° for a cistern orientation of α = 0°. In experiment 3 (Figure 5C) shaking was done with 10 rpm *in vitro* (corresponding to 2.5 rpm *in vivo*) and with a φ_max_ = 45° for a cisternal orientation of α = 0°. The corresponding curves are shown in Figure 6B. The shortest (best) clearance time of steady CSF net flow experiments was achieved with the settings of Exp. 1 (α = 0°, φ_max_ = 70°, shaking frequency *f* = 0.25 rpm *in vivo*).

## Discussion

Clot clearance from the basal cisterns after subarachnoid hemorrhage has been studied by means of serial computer assisted tomography (CAT) [4]. To our knowledge, there are no MRI-based clearance studies available. CAT or MRI can be used to assess clot clearance only in limited i.e. larger time intervals. We established an *in vitro* model to continuously study dynamics of clot clearance from the basal cistern. Furthermore, this model allows us to deduce suitable parameters for head shaking, a promising, non-pharmacological and non-invasive therapy to enhance subarachnoid clot clearance. *In vitro* blood clearance by steady CSF net flow in the basal cistern with and without head shaking was visualized by means of quantified dye washout. Dye washout is an established method used in clinical medicine (e.g. X-ray angiography) and in experimental and numerical flow visualization [19]–[21]. Our dye washout experiments indicate that cisternal blood clearance due to CSF net flow (20 ml/h) without additional external head motion (head shaking) is a very long lasting process (Figure 6A). In the basal cistern without blood, flow of CSF causes a relatively fast washout (Figure 6, CSF-washout curve). This can be explained by the fact that CSF flow is a creeping (Stokes) flow – a flow where inertial forces are small compared with viscous forces. With the given hydrodynamic diameter of the middle cross-section (10 mm depth and 56 mm width) the calculated Reynolds number equals 0.27.

**Figure 7:**
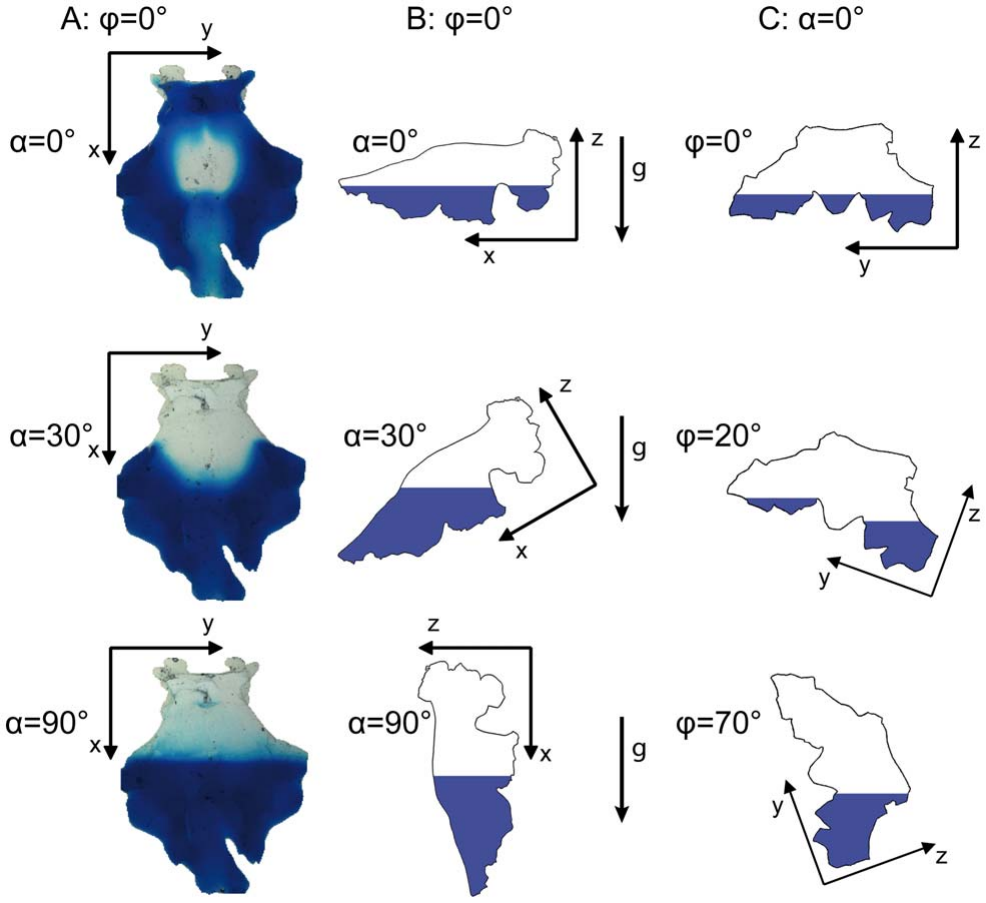
Scheme of the influence of gravitational alignment and shaking angle. Blood model distribution images (A) and schematic drawings (B) after injection (φ = 0°, T = 0 min). The g-arrows indicate the direction of the gravitational force, blue regions correspond to experimental blood, whereas white regions to experimental CSF. Different cistern orientations (α = 0°, 30°, 90°) cause different CSF-blood layers. (C) Schematic drawings of different shaking angles for a given cistern position (α = 0°). The complex shape of the basal cistern hampers extensive movement of cisternal blood if the shaking angle is too small.

The cisternal flow dynamics are fundamentally changed after SAH. Directly after a SAH, CSF and blood are essentially separated in the basal cistern due to differences in viscosity and density (see Movie S1). A split fluid-layer system forms with blood beneath the CSF (Figure 7A and B). Resistance of laminar duct flow is linearly proportional to viscosity. Therefore and since CSF has a kinematic viscosity of at least 3.5 times lower than blood, CSF flows round the cisternal blood via the paths of least flow resistance. Mixing of blood and CSF is required in order to increase the rate of blood clearance. Blood-CSF mixing due to diffusion corresponding to the blood-water diffusion process is very slow due the low diffusion coefficient of about D_BW_ = 0.6·10^−9^ m^2^/s [22], corresponding to a characteristic diffusion time (T_D_ =  R^2^/4 · D_BW_) of about 650 min for a distance of R = 10 mm. To visualize blood clearance, dyed experimental blood was used. Diffusion between dyed experimental blood and non-dyed experimental CSF differs from diffusion *in vivo* and may alter experimental results. However, as found by the “Blood-CSF-diffusion” reference experiment the diffusion coefficient of the used dye is very low. Therefore, the results of blood washout experiments simulating head shaking are not affected. Figure 6A includes the curve of Exp. 10 simulating experimental blood/CSF diffusion. There is only slow mixing without washout.

Our experiments revealed enhanced longitudinal washout after transversal mixing generated by head shaking. Blood clearance in a supine patient with slightly reclined head (α = 0°) increases with slower shaking frequencies and with higher shaking angles φ_max_ (Table 2).

We see several possible explanations for our experimental findings:

First, the mixing process is driven by gravitational force. Shaking changes the orientation of the basal cistern relative to the direction of earth’s gravitational force. The change in the direction of gravitational force causes movement (shifting) of the blood layer, which has a slightly higher density than CSF (ρ_CSF_/ρ_blood_ = 0.95). This motion is, however, very slow due to blood viscosity and the relatively small difference in density. Mixing by centrifugal acceleration due to shaking is negligible for the same reasons. Thus, slower shaking frequencies and greater shaking amplitudes are more suitable for therapeutical applications. These findings stand in contrast to therapy strategies in previous studies, in which lateral head movements with high frequencies of 0.5 to 3.0 and of 1.0 to 1.5 cycles per second respectively [7], [8] and a small translational amplitude of 4 cm were used [7]. Kawamoto et al. compared two groups of patients with SAH with both groups receiving cisternal washing via external catheters [7]. Only one group underwent additional translational low amplitude shaking. The incidence of a permanent neurological deficit was reduced by shaking (significance level p of 0.061). Furthermore, interpretation of the effect of shaking is obscure because in both groups cisterns were irrigated with a solution containing urokinase. Nakgomi et al. compared two groups of patients with SAH, one treated with cisternal washing and shaking and the other one treated without these measures [8]. The group with cisternal washing and head shaking had a significantly (p<0.05) decreased incidence regarding cerebral infarction, mortality, and morbidity due to vasospasm compared to the group without shaking and cisternal irrigation. Therefore, the significant better results in the group with shaking might been caused by cisternal washing and irrigation with urokinase. Hänggi et al. compared a study group of patients with surgically obliterated aneurysms and treated with rotational head-motion combined with lumboventricular lavage and a control group of patients whose aneurysms had been endovascularly obliterated and who were treated without rotational head motion or lumboventricular lavage [9]. Thus the statistically positive outcome in the study group could also been attributed to the effect of cisternal irrigation during and after surgery and not to the rotational head motion. So far no study has been published in which head shaking alone is compared with conventional treatment without cisternal irrigation.

Second, the complex shape of the basal cistern containing cavities and regions of convex and concave surfaces (Figure 7) may prevent movement of cisternal blood. With too small shaking angles (e.g. φ_max_ = 20°, Figure 7C), one would expect no increase in mixing and hence no improvement of blood washout. Consequently, larger shaking angles remarkably improve washout (Table 1 and Figure 7C, φ_max_ = 70°).

Third, we observed variations in blood distribution after injection due to the cistern orientation (Figure 5 and 7 and Movie S1). Location of blood in the basal cistern might have an impact on the mixing process. In our study, we did not vary the location of the bleeding source, what might also affect location and distribution of the cisternal blood.

Our findings allow general statements and are independent of individual differences in geometry and blood viscosity, which of course might have an impact on the patient-specific effectiveness of shaking therapy and the outcome after cisternal bleeding.

### Study Limitations

This study has limitations. Blood is a non-Newtonian fluid [23], a feature altering its characteristics due to the coagulation process in the CSF. However, sparse information is available about blood rheology in the CSF after a SAH. Naff et al. found a first-order kinetic of the blood clot resolution, which was independent from clot volume, age, sex and use of external drainage [24]. They noted also that the thrombolytic enzyme system seems to be saturated at 24–48 hours after SAH. An increased blood viscosity due to coagulation is expected to hamper blood/CSF mixing and hence blood clearance. On the other hand, naturally occurring thrombolysis results in a lesser difference between viscosities and densities of blood and CSF and would facilitate blood clearance. This might imply that a therapeutic intervention aiming a removing of cisternal blood should start within the first 48 hours after SAH.

Our anatomical model is based on MRI data from a 35-year-old male whereas the average male patient with a ruptured aneurysm is about 50 years old (49.4±12.2 mean±s.d., CLARITY study [25]). Furthermore there is an increase in total subarachnoid volume with age [26]. This age-dependent increase in total subarachnoid volume results in a decrease of CSF clearance rate [27] thus challenging subarachnoid blood clearance. This emphazises the importance of active subarachnoid clearance mechanisms as head shaking and increased CSF perfusion with or without cisternal thrombolysis. Nevertheless our study does not provide statistical data on the age-dependency but on basic mechanisms of subarachnoid clearance in head shaking.

We studied only the basal cistern and not the adjacent anterior and lateral cisterns. These more peripheral cisterns empty into the basal cistern. Therefore, unplugging the basal cistern by clearing it from blood enables a better clearance of the peripheral cisterns.

The patient-specific anatomic model based on MRI measurements does not incorporate arachnoid membranes. Due to their delicate structure still elusive to modern imaging, these membranes can be modeled only in numerical studies assuming the basal cavity as a porous volume. To the best of our knowledge, only one such computational fluid dynamics (CFD) study of CSF flow in the subarachnoidal space using an anisotropic porous model has been published [15]. A CFD study simulating blood clearance over many hours is possible but requires enormous computational time. One would expect that anisotropy of permeability coefficients (factor six higher in a longitudinal direction [15]) would hamper the transversal mixing process. But due to the very high porosity of 0.99 [28], the existence of arachnoid membranes does not affect significantly CSF flow dynamics and hence our findings. This hypothesis is supported by the good correlation between measured CSF flows and simulations using models without arachnoid membranes [29]. The impact of arachnoid membranes, which are known to increase the static pressure in the basal cistern [15], on CSF dynamics should be, however, investigated more detailed in a separate CFD study.

In our study, we model steady CSF net flow. Magnetic resonance imaging measurements showed the pulsatile nature of CSF flow with high flow rate amplitudes during the heart cycle [15]. Actually, CSF flow changes its direction during the heart cycle. MRI-measurements in the pontine cistern found a maximal positive flow rate of 100 ml/min and a maximal negative flow rate of 50 ml/min with an *in vivo* net CSF flow rate of only 20 ml/h. CSF flow oscillating with the heart rate also increases CSF/blood mixing and thereby blood clearance. In Figure 6B, the curve labeled with “pulsation” describes the blood washout in the experiment with pulsatile CSF flow, a cistern orientation of α = 0° and without shaking (Exp. 11). The effect is even slightly better than our best washout experiment (Exp. 1) with steady net CSF flow and shaking. The clearance time T_C_ of 23 min was 30% shorter than T_C_ of Exp. 1 (Table 2). Since the aim of our study was to analyze the effect of head shaking apart of other factors, the pulsatile nature of CSF flow was not considered in the first step. Consequently, the majority of the washout experiments were performed assuming natural net CSF flow. Note that a study of shaking therapy under pulsatile CSF flow conditions is challenging. Head shaking may affect volumes of three compartments with CSF, arterial and venous blood, which define the CSF flow pulsatility [24] and in turn affect the CSF pulsatility by increasing or decreasing of the pulsation amplitude. This may hamper or facilitate blood clot clearance.

Increased CSF flow seems to be advantageous in the treatment of SAH [5]. In any case, increased CSF flow enhances subarachnoid blood clearance. The existence of a mixing effect due to head shaking is, however, independent from CSF flow rate or pulsation.

### Conclusions

Quantitative dye visualization was used to study *in vitro* the effects of head shaking on blood clearance in a model of cisternal SAH. Our experimental study supports the clinical assumption that head shaking can increase the subarachnoid blood clearance significantly. Our results provide insights into the effects of different shaking strategies (with respect to shaking frequency and angle amplitude) upon the washout process in a model of the basal cistern.

Based on the results, we expect that head shaking with low shaking frequencies and high shaking angles (amplitudes) will improve cisternal blood clearance. This should be, however, tested in a clinical study.

Open questions include the influence of pulsatile CSF flow known from MRI measurements [15], the effect of increased CSF flow (simulating cisternal irrigation), and the implication of increased blood viscosity (simulating a delayed start of the shaking intervention). Experimental answers to these questions might refine a treatment protocol for cisternal SAH combining head shaking and cisternal irrigation with or without a thrombolytic agent.

### Key Messages

- the hypothesis that head shaking is a therapeutic strategy to increase blood washout in the subarachnoid space is supported by the presented experimental *in vitro* study
- blood washout of the subarachnoid space consists of two distinct phases: Blood/CSF mixing and clearance
- head shaking facilitates blood washout by varying the direction of gravitational force
- blood washout increases with decreased shaking frequency and with increased shaking angle amplitude

## Supporting Information

Movie S1 **Injection of experimental blood.** Experimental blood injections into the model cistern simulating a supine patient with slightly reclined neck and a supine patient with normal head position.
(AVI)

Movie S2 **Experimental blood washout.** Washout of the experimental blood with the experimental setting Exp. 1.
(AVI)
